# Sudden Cardiac Arrest With Ventricular Fibrillation in a Patient With Epilepsy and a Vagus Nerve Stimulator

**DOI:** 10.7759/cureus.86182

**Published:** 2025-06-17

**Authors:** Abdelhalim Eltaib, Ahmed Abdelmageed, Aftab Gill

**Affiliations:** 1 Cardiology, Queen's Hospital Burton, Burton, GBR; 2 Internal Medicine, Queen's Hospital Burton, Burton, GBR

**Keywords:** automated external defibrillator (aed), epilepsy, postictal arrhythmia, seizure, sudden cardiac arrest, vagus nerve stimulator, ventricular fibrillation

## Abstract

Sudden cardiac arrest (SCA) in patients with epilepsy, particularly those with vagus nerve stimulators (VNS), is rare but clinically significant. We report the case of a 36-year-old woman with known epilepsy and a VNS implant who suffered an out-of-hospital cardiac arrest (OHCA) following a witnessed tonic-clonic seizure. Emergency services identified ventricular fibrillation (VF), and successful defibrillation led to the return of spontaneous circulation (ROSC). Initial investigations, including imaging and laboratory studies, were unremarkable. A multidisciplinary evaluation involving neurology and cardiology was undertaken to explore potential seizure-related cardiac mechanisms and the possible role of the VNS device. This case highlights the complex interplay between epilepsy, autonomic dysfunction, and arrhythmia, emphasising the importance of integrated care and further research into neurocardiac interactions in patients with epilepsy.

## Introduction

Seizures are characterized by abnormal electrical activity in the brain, which can trigger a cascade of physiological responses, including cardiac arrhythmias. Postictal arrhythmias may arise from mechanisms such as vagus nerve overactivity, respiratory compromise, catecholamine surge, abnormal cardiac repolarization, and the pharmacodynamic effects of antiepileptic drugs. These autonomic and electrophysiological disturbances can, in rare instances, lead to life-threatening cardiac events. The association between epilepsy and sudden cardiac arrest (SCA) is increasingly recognized, although the precise pathophysiological mechanisms remain incompletely understood [1-3].

One of the most devastating epilepsy-related complications is sudden unexpected death in epilepsy (SUDEP), defined as a sudden, non-traumatic, and non-drowning death in a person with epilepsy, with no structural or toxicological cause identified postmortem. SUDEP is the leading cause of death in patients with refractory epilepsy, with estimated incidence rates ranging from 0.1 to 1 per 1,000 patient-years in the general epilepsy population, and higher rates in those with uncontrolled seizures.

This case report describes a patient with epilepsy and an implanted vagus nerve stimulator (VNS) who experienced a witnessed postictal out-of-hospital cardiac arrest (OHCA) due to ventricular fibrillation (VF). VNS is widely accepted as an effective adjunctive therapy for drug-resistant epilepsy, modulating neural activity via afferent stimulation of the vagus nerve. Although generally well tolerated, VNS has been associated in isolated reports with cardiac arrhythmias, particularly bradyarrhythmias, asystole, and, very rarely, ventricular arrhythmias such as VF [4-8]. The combination of antiepileptic drugs with other QT-prolonging medications, such as selective serotonin reuptake inhibitors (SSRIs), may further increase the risk of malignant arrhythmias [2,9,10].

Sudden cardiac arrest in patients with epilepsy presents significant diagnostic and therapeutic challenges. The intersection of seizure activity, cardiac instability, and device-based therapy necessitates a multidisciplinary approach, involving neurology, cardiology, and electrophysiology, to ensure accurate diagnosis, comprehensive risk assessment, and individualized management [5,6].

## Case presentation

**Figure 1:**
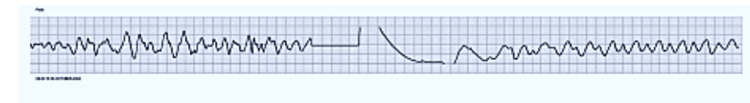
Ambulance ECG rhythm strip obtained from the automated external defibrillator (AED) download, demonstrating the onset of ventricular fibrillation. Chaotic, irregular, high-frequency waveform consistent with ventricular fibrillation (VF). No alterations were made to the diagnostic features of the ECG tracing.

A 36-year-old woman with a known history of epilepsy, managed with sodium valproate, lamotrigine, clonazepam, and a vagus nerve stimulator, as well as comorbid anxiety and depression treated with citalopram, was brought to the emergency department by ambulance following an OHCA. The event was preceded by a generalised tonic-clonic seizure at home, witnessed by her daughter, who promptly called emergency medical services. Upon arrival, paramedics found the patient pulseless with agonal respirations, and automated external defibrillator (AED) analysis revealed VF (Figure 1). A single shock was delivered, resulting in return of spontaneous circulation (ROSC), after which the patient was transported to the hospital for further evaluation and management.

Hospital course

**Figure 2:**
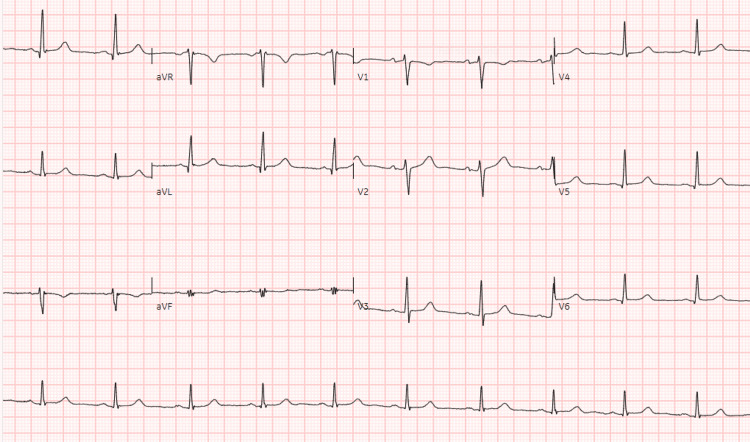
12-lead electrocardiogram (ECG) post resuscitation showing a normal sinus rhythm. 12-lead ECG post resuscitation demonstrating a normal sinus rhythm, with a heart rate of approximately 75 bpm, PR interval ~160 ms, QRS duration ~90 ms, and QTc ~430 ms. No ST-segment abnormalities or T wave changes are observed.

Upon arrival at the emergency department, the patient was intubated for airway protection and admitted to the intensive care unit (ICU) for close monitoring. After two days in the ICU, her condition stabilised, and she was transferred to the coronary care unit (CCU). Initial investigations, including electrocardiogram (ECG), transthoracic echocardiogram, and computed tomography (CT) scans of the chest and head, were unremarkable. Post-resuscitation ECG showed normal sinus rhythm, and there was no evidence of acute ischemia or structural abnormality (Figure 2).

**Figure 3:**
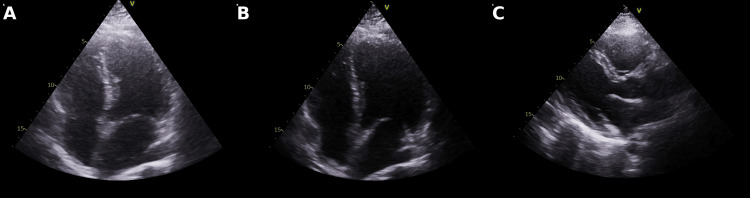
Echocardiographic views. A: Apical four-chamber view during systole showing normal contraction of the left and right ventricles. B: Apical four-chamber view during diastole illustrating ventricular filling with clearly visualized left and right atria. C: Parasternal long axis view depicting normal morphology of the left ventricle, mitral valve, and aortic valve.

Transthoracic echocardiography confirmed preserved left ventricular systolic function (LVEF >55%), normal diastolic function, and no significant valvular pathology (Figure 3).

A neurology consultation was obtained to assess the potential contribution of epilepsy to the cardiac arrest. Although the VNS device was not formally interrogated at the time of the event, a thorough review was conducted in collaboration with the treating neurologist and a VNS-specialized nurse. No evidence of device malfunction, inappropriate stimulation, or parameter deviations was identified during the event or subsequent follow-up assessments.

**Figure 4:**
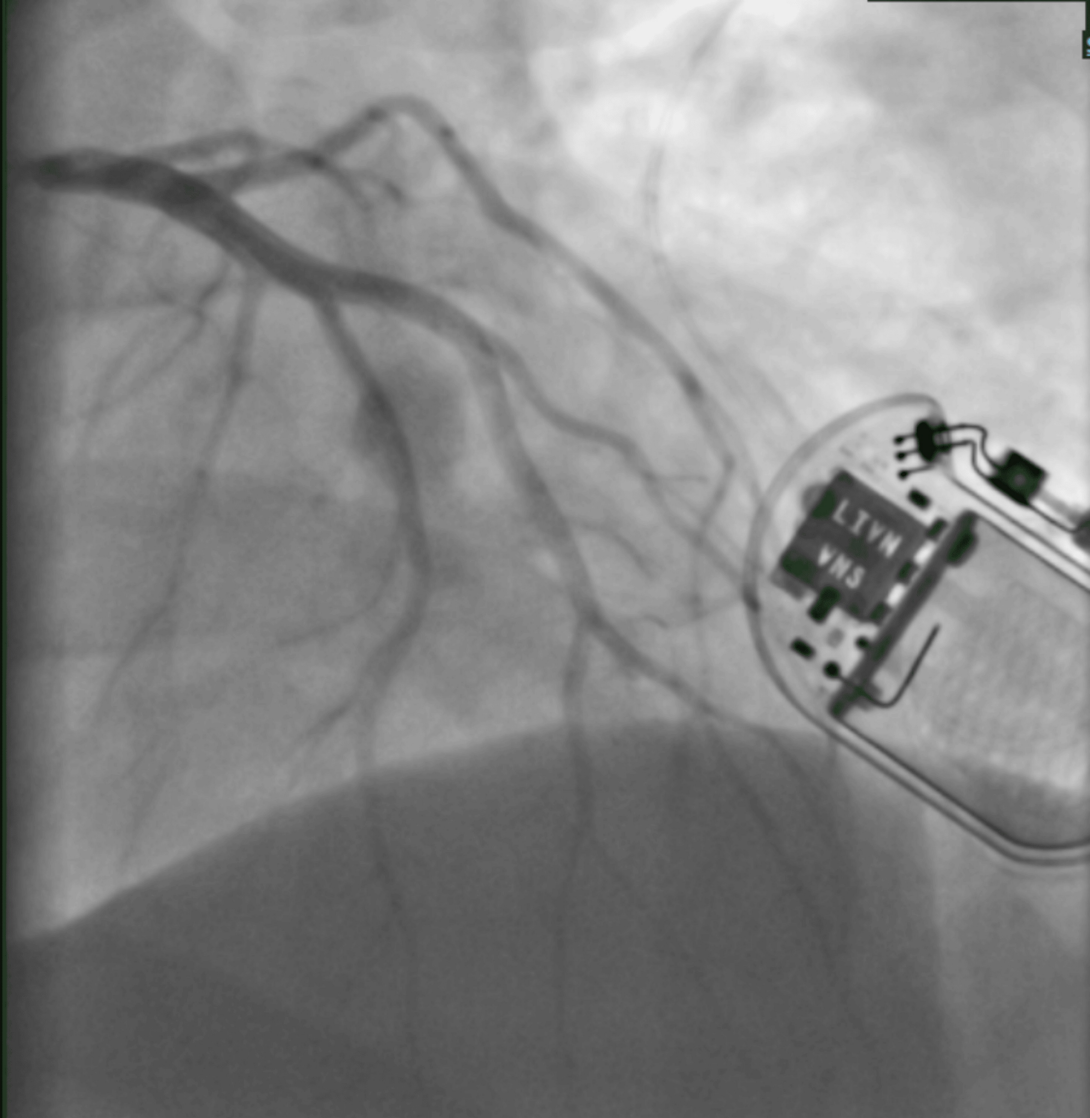
Left coronary angiography. Normal left coronary artery system with no evidence of luminal narrowing, atherosclerotic changes, or flow-limiting lesions.

**Figure 5:**
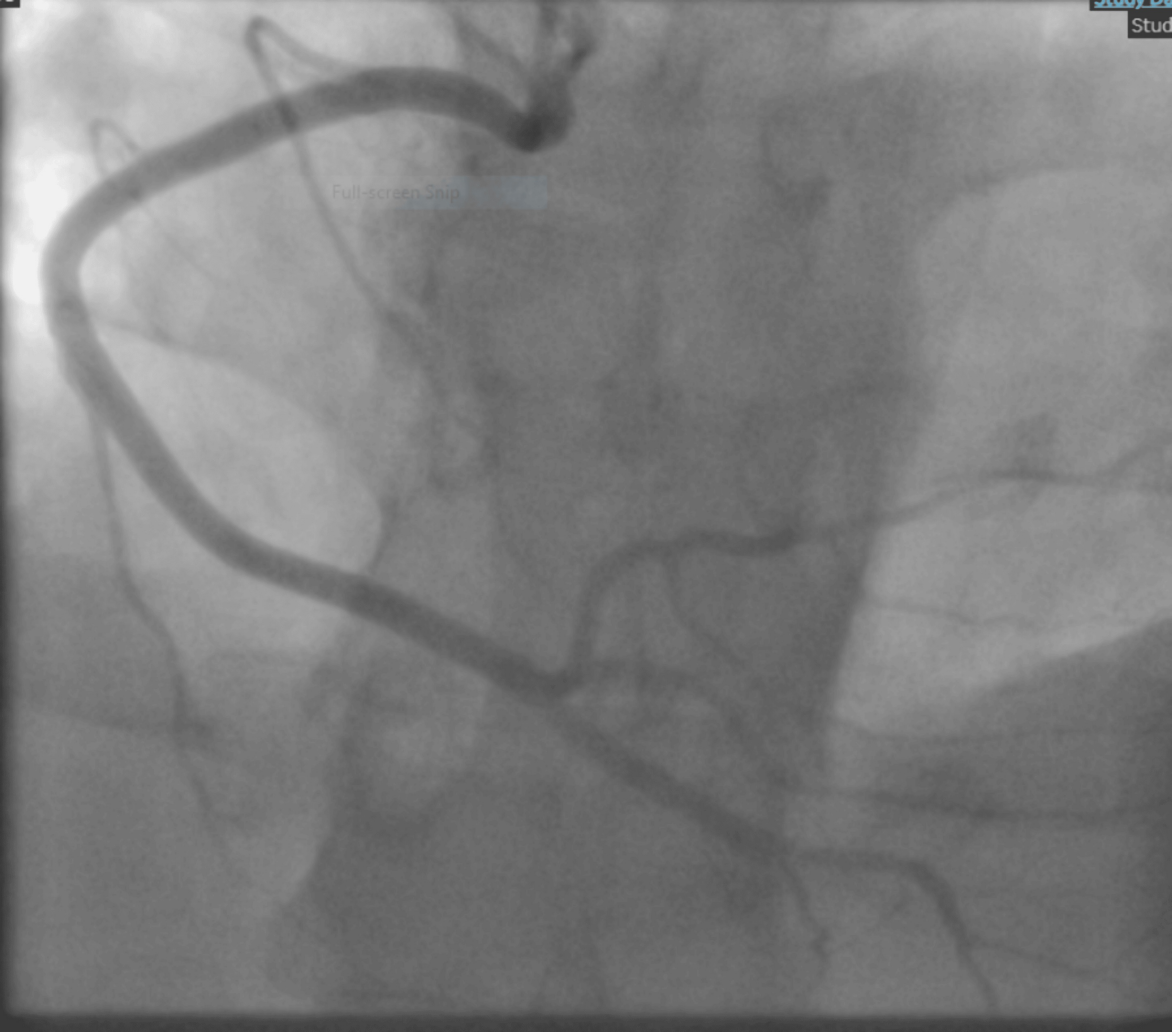
Right coronary angiography. Normal right coronary artery with unremarkable flow and no signs of stenosis or irregularities.

A diagnostic coronary angiogram was performed and demonstrated normal coronary arteries without any flow-limiting lesions (Figures 4-5).

Cardiac magnetic resonance (CMR) imaging was planned to further evaluate for potential structural or infiltrative cardiac disease; however, it was deferred due to concerns regarding MRI compatibility with the patient's implanted VNS.

**Figure 6:**
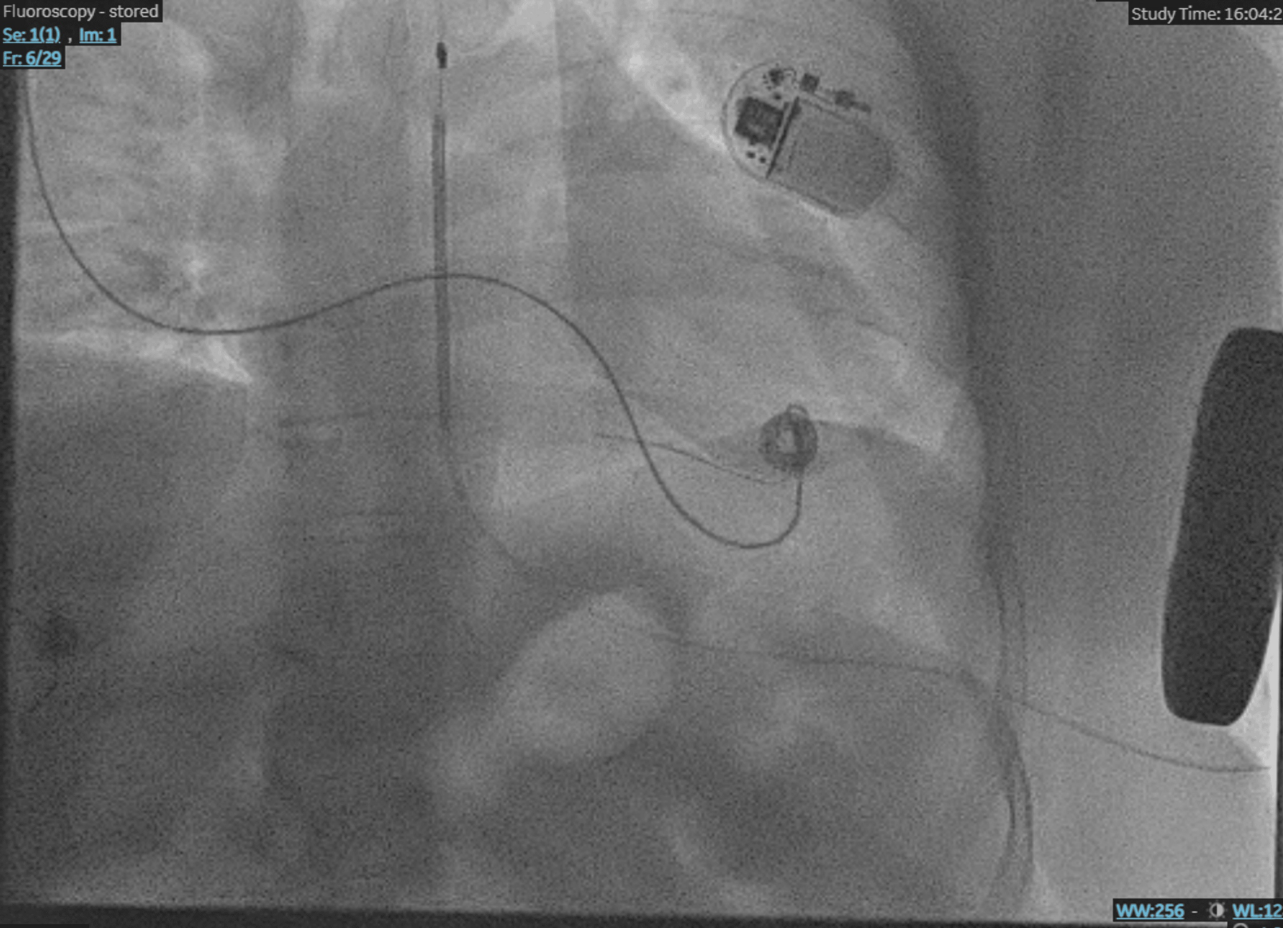
Subcutaneous implantable cardioverter defibrillator (S-ICD). Post-implantation chest image confirming the correct placement of the subcutaneous ICD, indicated for secondary prevention following a ventricular fibrillation episode.

Given the occurrence of VF with successful resuscitation and no reversible cause identified, a multidisciplinary cardiology team recommended implantation of a subcutaneous implantable cardioverter-defibrillator (S-ICD) for secondary prevention. The choice of an S-ICD was made to avoid complications associated with transvenous leads and to account for the potential impact of seizure-related motion artifacts on device sensing and therapy delivery. The ICD was successfully inserted prior to hospital discharge (Figure 6).

Follow-up and imaging considerations

Upon discharge, the patient was planned to be followed up closely by the cardiac electrophysiology team, who would assess the clinical course and determine the need for further imaging. If indicated and deemed safe, an MRI would be pursued. Alternative imaging modalities, such as CT or positron emission tomography (PET), would also be considered appropriate to guide ongoing evaluation and management.

## Discussion

This case underscores the potential for serious cardiac complications in patients with epilepsy, particularly the rare but life-threatening occurrence of SCA. While the precise aetiology of the cardiac arrest in this patient remains uncertain, several plausible mechanisms merit consideration, reflecting the intricate interplay between neurological pathology and cardiac function.

Although SUDEP is a recognized and serious complication of epilepsy, this case does not meet the classical SUDEP criteria, as the cardiac arrest was witnessed, a VF rhythm was documented, and the patient survived following prompt resuscitation. Nevertheless, the event highlights overlapping mechanisms of seizure-related autonomic instability and SCA, warranting continued interdisciplinary surveillance [2].

Autonomic dysregulation

Cardiac autonomic dysfunction is a well-recognized phenomenon in epilepsy. Seizures, especially generalized tonic-clonic seizures, can disrupt autonomic balance, affecting both sympathetic and parasympathetic outputs. Sympathetic overactivity, often presenting with tachycardia, hypertension, and diaphoresis, is commonly observed during seizures. Conversely, parasympathetic activation may induce bradycardia and hypotension. These autonomic shifts can occur independently or simultaneously, potentially contributing to significant cardiovascular instability during the peri-ictal and postictal periods. In some cases, this dysregulation may facilitate arrhythmogenesis and precipitate sudden cardiac events, including SCA and SUDEP [11].

Drug-induced effects

Pharmacological factors also warrant consideration. Several antiepileptic drugs, notably lamotrigine, carbamazepine, and gabapentin, exert sodium channel-blocking effects that may influence cardiac conduction. These effects can manifest as QT interval prolongation, PR prolongation, or bradyarrhythmias, all of which may predispose patients to life-threatening ventricular arrhythmias, such as torsades de pointes or VF. This risk may be compounded by concurrent use of other QT-prolonging medications, such as selective serotonin reuptake inhibitors (SSRIs) [2,3].

In this case, the patient was receiving lamotrigine and sodium valproate. While these agents are generally considered to have low arrhythmogenic potential in therapeutic doses, lamotrigine has been implicated in rare cases of cardiac conduction abnormalities. The patient was also taking citalopram, a well-established QT-prolonging SSRI. However, the electrocardiogram (ECG) obtained during evaluation showed a normal QT interval, and no conduction abnormalities were observed. Although pharmacologic contributions cannot be entirely excluded, the absence of ECG changes suggests that the patient’s medication regimen was unlikely to be the primary cause of the event. Nevertheless, the potential role of these agents was considered in the differential diagnosis.

VNS device-related influences

The presence of a VNS introduces additional complexity. VNS is widely regarded as a safe and effective adjunctive treatment for drug-resistant epilepsy. However, isolated reports have described its association with cardiac arrhythmias, most notably bradyarrhythmia-induced syncope, asystole, and atrioventricular (AV) conduction abnormalities. These events are rare and often occur in susceptible individuals, typically within the first few days after device implantation or during stimulation threshold titration.

In this case, the patient experienced VF, a malignant arrhythmia not commonly linked to VNS in the literature. Given the absence of more typical arrhythmias such as bradycardia or AV block, the role of VNS remains speculative. The device was not formally interrogated, but it was thoroughly reviewed with the treating neurologist and a VNS-specialized nurse. No concerns were raised regarding device malfunction, inappropriate stimulation, or parameter deviations during the event or follow-up visits.

Interestingly, some emerging evidence suggests that cardiac-directed VNS may reduce seizure burden and affect cardiac autonomic tone, highlighting a complex and potentially bidirectional interaction between the autonomic nervous system and seizure activity. Nonetheless, more robust research is needed to understand the conditions under which VNS may exert protective versus proarrhythmic effects. In this case, while a definitive link between VNS and VF cannot be established, it remains a consideration in the differential diagnosis, especially in the context of overlapping autonomic and neurological vulnerabilities [1,4-8].

Diagnostic challenges

The presence of an implanted VNS device also introduces diagnostic limitations. In this case, CMR imaging, a valuable modality for detecting structural or infiltrative myocardial pathology, was deferred due to concerns about MRI compatibility. This limitation may have impeded a full evaluation for underlying cardiomyopathy or myocardial fibrosis, which are known substrates for ventricular arrhythmias [6].

While newer MRI-conditional VNS systems are now available, the patient’s device did not meet these criteria. Although reprogramming strategies to facilitate safe imaging were considered, the multidisciplinary team - including neurology and cardiology - advised against performing MRI during the acute evaluation due to safety concerns.

Furthermore, population studies indicate that individuals with epilepsy have a significantly elevated risk of SCA compared to the general population, even outside the context of active seizures. Recent findings from a large cohort study confirm that epilepsy confers an increased long-term risk of arrhythmias, independent of other comorbidities [3,7,11].

## Conclusions

This case highlights the diagnostic and therapeutic challenges arising from the intersection of epilepsy and cardiovascular pathology, particularly in the setting of VNS. Although ventricular fibrillation in this context is rare, its potentially life-threatening severity underscores the critical need for a multidisciplinary approach involving neurology, cardiology, and electrophysiology to comprehensively assess and manage patients with epilepsy and VNS who experience unexplained cardiac events.

Future research should prioritize the systematic investigation of the proarrhythmic potential of VNS, explore the interactions between antiepileptic drugs and VNS on cardiac electrophysiology, and focus on developing robust risk-stratification tools to better predict and prevent sudden cardiac events in patients undergoing neuromodulation therapy.
